# Screening of bioflocculant and cellulase-producing bacteria strains for biofloc culture systems with fiber-rich carbon source

**DOI:** 10.3389/fmicb.2022.969664

**Published:** 2022-11-24

**Authors:** Jinping Wu, Yifeng Chen, Xueni Xu, Wei Ren, Xiang Zhang, Xiaoni Cai, Aiyou Huang, Yanhua Zeng, Hao Long, Zhenyu Xie

**Affiliations:** ^1^Hainan Provincial Key Laboratory for Tropical Hydrobiology and Biotechnology, Hainan University, Haikou, Hainan, China; ^2^College of Marine Sciences, Hainan University, Haikou, Hainan, China; ^3^State Key Laboratory of Marine Resource Utilization in the South China Sea, Hainan University, Haikou, Hainan, China; ^4^Laboratory of Development and Utilization of Marine Microbial Resource, Hainan University, Haikou, Hainan, China

**Keywords:** biofloc, sugarcane bagasse, *Litopenaeus vannamei*, bioflocculant, cellulase

## Abstract

The biofloc technology (BFT) system has been widely applied in the shrimp and fish culture industry for its advantages in water-saving, growth improvement, and water quality purification. However, The BFT system usually takes a long time to establish, and the extra carbon source input increases the maintenance cost of the system. In this study, we aimed to develop a low-cost and high-efficient BFT system for *Litopenaeus vannamei* by applying bacteria that could promote the formation of BFT and utilize cheap carbon sources. Three bioflocculant-producing bacteria strains (M13, M15, and M17) have been screened from a cellulolytic strain collection. All three strains have been identified as *Bacillus spp*. and can use sugarcane bagasse (SB) as a carbon source, which is a cheap byproduct of the sucrose industry in the tropic area of China. Compared to sucrose, the addition of SB and the three strains could improve the biofloc formation rate, biofloc size distribution, ammonia removal rate, and the growth performance of the shrimps. These results suggest that the bioflocculant and cellulase-producing bacteria strains could promote the biofloc formation and the growth of shrimps by using SB as an economic substitute carbon source in the BFT shrimp culture system.

## Introduction

In China and many other areas with a large populations, aquaculture developed rapidly to provide a cheap source of protein (Boyd et al., 2022). The pacific white shrimp (*Litopenaeus vannamei*) has been widely comsume as an excellent resource for its high-protein and cholesterol meat (Pan et al., 2019). With the development of the aquaculture industry, a large amount of water is required to eliminate the waste produced by the farmed animals, which consumed clean water resources and polluted the environment (Lebel et al., 2019; Jayanthi et al., 2020). In aquaculture or other eutrophic water systems, microorganisms such as bacteria, algae, and protozoans can combine with organic debris to form particles which are termed biofloc (Jamal et al., 2020). By converting the nitrogenous wastes into microbial biomass, biofloc technology (BFT) was applied in the aquaculture system to keep the water quality without a large-scale water change and the biofloc particles could also be taken by shrimps or fish as additional nutrition (Santhana Kumar et al., 2018; Putra et al., 2020; Van Doan et al., 2021). In shrimp culture system, studies suggested that biofloc can enhance the growth and the immune response of shrimps by altering the bacterial communities and reusing the waste in the water (Jamal et al., 2020; Addo et al., 2021; Panigrahi et al., 2021; Xu et al., 2022).

Bioflocculants are composed of biodegradable polymers, such as glycoprotein, polysaccharide, protein, lipids, and nucleic acid that are produced by growing microorganisms (Lian et al., 2008; Liu et al., 2014). Bioflocculants were widely used in wastewater treatment through their ability to aggregate suspended solids together and adsorb heavy metals without causing secondary pollution for the environment (Liu et al., 2020; Vimala et al., 2020; Tsilo et al., 2022). Certain bioflocculant-producing bacteria possessed the antagonistic ability to pathogenic *Vibrio* species, which could be used to control *Vibrio* infection and apply as inoculum for bioflocculation simultaneously (Tuan et al., 2021). Also, adding bioflocculant-producing bacteria into the aquaculture system could promote the formation of biofloc (Luo et al., 2016; Kasan et al., 2017; Jiang et al., 2019).

Another key element of the biofloc system’s successful formation is the carbon/nitrogen ratio (C/N). In regular aquaculture systems, inorganic nitrogen concentration accumulates rapidly due to high protein feeds input and the C/N ratio is too low to support heterotrophic bacteria and the formation of biofloc (Avnimelech, 1999). Therefore, an extra carbon source must be added to the water system to reach an optimal C/N ratio for the biofloc to remove residual nitrogen and recycle nutrients (Tayyab et al., 2021). However, an extra carbon source also means a rise in the cost, which is not acceptable since the limited profit of common farmed fish and shrimps. A low-cost carbon source suitable for the biofloc system is urgently needed in this case. Sugarcane bagasse (SB) is the major waste product of the sugar industry, especially in tropic areas where sugarcane is the main source of sucrose (Dong et al., 2017). Composed mainly of cellulose (35%–50%), hemicellulose (20%–35%), and lignin (10%–25%), which are difficult to degrade, SB does not have large-scale applications in industry and has a low market price (Mahmud and Anannya, 2021; Shabbirahmed et al., 2022). There have been several applications of SB in wastewater treatment, which mainly used SB as a biosorbent to remove dyes, Pb2+, and ammonia from water (Krishnani et al., 2006; Aruna et al., 2021; Hoang et al., 2021).

In this study, our purpose is to screen bioflocculant-producing bacteria strains with the ability to use sugarcane bagasse as the main carbon source in shrimp culture factories. The bioflocculant produced by these strains will facilitate the biofloc formation and the ability to use with a low-cost carbon source could lower the maintenance cost of the biofloc shrimp culture system.

## Materials and methods

### Shrimps

Pacific White Shrimp, *Litopenaeus vannamei*, were provided by Hainan Zhongzheng Aquatic Science and Technology Co., LTD. Before the experiments, shrimps were domesticated for 1 month with continuous water exchange and constant aeration to adapt to the environment and feed. The shrimps were fed with commercial feed (Guangdong Evergreen Conglomerate Co., LTD., Guangdong, China) with a crude protein level of 42%. The feeding levels accounted for 3% of the shrimp’s body weight every day. The feces and feed residues at the bottom were removed and 30% of the rearing water in each tank was replaced daily.

### Strains

Strains with potential cellulolytic activity were selected as experimental candidates from the Microorganisms Collection of South China Sea, MCSCS, constructed in the previous work of our laboratory (Ren et al., 2021).

### Analysis of flocculating efficiency

The flocculating efficiency of the bioflocculant produced by the bacterial culture was measured using kaolin clay suspension (Luo et al., 2016). In general, 2 ml of the culture broth, 2 ml of CaCl_2_ (10 g/L), and 46 ml of kaolin clay suspension were mixed in a 100-ml beaker. The mixture was stirred at 250 r/min for 1 min and 60 r/min for 3 min with a vortex mixer and then kept still at room temperature for 5 min. The supernatant was sampled 2 cm below the surface and measured *via* spectrophotometry at 550 nm. The control was similar to the above steps except that the fermentation broth was replaced with an uninoculated culture medium. All assays were conducted in three duplicates.

The flocculating activity was calculated as follows:


Flocculating activity(%)=(A−B)/A×100

where A and B represent the OD of the control and real samples, respectively.

To verify the stability of the flocculant-producing ability of each strain, the top 10 strains of high flocculating activity were continuously cultured and tested for 3 generations.

### 16S rDNA analysis

The selected 3 flocculant-producing bacteria isolates were identified by molecular genetic analysis. The strains were cultured in broth for 16 h, then the cells were harvested and subjected to genome DNA extraction by a DNA extraction kit (Tiangen Biotech (Beijing) Co., LTD., Beijing, China). The universal primers of 27F (5′-GAGTTTGATCATGGCTCAG-3′) and 1492R (5′-CGGTTACCTTGTTACGACTT-3′) were utilized to amplify the 16S rRNA gene fragments. Polymerase chain reaction (PCR) was performed in a 50 μl reaction system containing 22.5 μl Green Taq Mix, 22.5 μl double-distilled water, 2 μl upstream primer, 2 μl downstream primer, and 1 μl DNA template. The PCR amplification was performed as follows: initial denaturation at 95°C for 5 min; 30 cycles of 94°C for 1 min, 50°C for 20 s, and 72°C for 2 min; and final extension at 72°C for 10 min. Bacterial sequences were compared with 16S rDNA reference gene sequences by BLAST.

### Biolog GEN III MicroStation system assay

The Biolog MicroStation System and GEN III microplate (Biolog Inc., Hayward, CA, United States) is an automated microbial identification system based on aerobic metabolic activities. The GEN III plate contains 95 different carbon substrates based on interpreting patterns of sole carbon substrate utilization indicated by color development in a 96-well microtiter plate. By analyzing the similarity of the metabolic fingerprints between test strains and standard strains in the kinetic database by Biolog software, the strains are identified. In this study, the strains M13, M15, and M17 were first cultured on BUG agar (provided by Biolog) and inoculated into a GEN III plate. After being cultured at 30°C for 24 h, the plate was read by a Biolog MicroStation reader to generate strain identification (Wozniak et al., 2019).

### Cellulolytic activity assay

Confirming of the cellulolytic bacteria was conducted by carboxymethylcellulose (CMC) agar plate (0.2% NaCl, 0.5% CMC sodium salt, 0.67% Na_2_HPO_4_, 0.13% (NH_4_)_2_SO_4_, 0.05% MgSO_4_·7H_2_O, and 1.7% agar) and Congo red staining method (Ma et al., 2020). The hydrolysis capacity (HC) value, which determined the enzymatic activity, was calculated from the ratio of clear zone diameter over the colony zone diameter.

Endoglucanase (CMCase) and filter paper activity (FPase) were used to estimate the cellulolytic activity, which was measured by the dinitrosalicylic acid (DNS) method using glucose as the standard (Azkawi et al., 2018).

The endoglucanase activity was determined by measuring the released reducing sugar. The seed bacteria were inoculated at a ratio of 1% (v/v) into CMC broth (0.2% NaCl, 0.5% CMC sodium salt, 0.5% tryptone, and 0.1% yeast extract) at 30°Cfor 24 h under 180 rpm. The cell-free supernatant was obtained by centrifugation (5,000 rpm, 4°C, and 15 min) to examine the activities of crude cellulase. Briefly, 1.5 ml CMC-Na solution and 0.5 ml enzyme solution were added to a 25 ml test tube at 50°C for 30 min. The enzyme reaction was terminated by adding 1.5 ml of DNS reagent and then boiled for 5 min. The optical density of the reaction mixture was measured at 540 nm.

The FPase activity was tested by incubating 0.5 ml of the supernatant diluted in 1 ml buffer solution of sodium citrate (100 mM, pH 4.8) with a filter paper strip of 1.0 × 6.0 cm (≈50 mg) for 1 h at 50°C. The color development process and determination were consistent with the CMC enzyme activity determination method.

In this experiment, the gravimetric method was used to determine the decomposition rate of Sugarcane bagasse (SB). Firstly, 1.25 ml of the fresh cultured bacterial solution was transferred to conical bottles containing 25 ml medium with 0.1 g bagasse. While the medium without bacteria was used as blank control, and all groups were cultured at 30°C and 180 r/min for 3 days. On the third day, a 7 ml neutral washing solution was added to the samples and then sterilized. After cooling to room temperature, the samples were poured into the crucible of the core which was dried in advance, and the water was filtered by the filtration pump. Then, 10 ml of absolute ethanol and 95% ethanol were added in sequence. Finally, the crucible with the sample was dried to constant weight and weighed.

The decomposition rate of SB of 3 strains was calculated according to the following formula:


$$\mathrm{Decomposition rate}\;\left(\%\right)=\frac{X-(W_{2}-W_{1)}}{X}\times 100\%$$

where X is the weight of the original SB; W_1_ is the constant weight of the sand core crucible; W_2_ is the constant weight of sand core crucibles with remained SB.

### Biofloc culture system

The shrimp biofloc culture system was carried out using 200 L circular tanks. Each tank was filled with 100 L seawater and reared with *L. vannamei*. The shrimp’s initial body weight and body length were 0.81 ± 0.08 g and 4.33 ± 0.45 cm, respectively.

The biofloc protocols were adopted BFT shrimp culture studies with some modifications (Santhana Kumar et al., 2018; Panigrahi et al., 2020; Magana-Gallegos et al., 2021). A total of six treatment groups were set up in the experiment, as shown in Table 1. The shrimp feeds containing ~40% protein was added into each tank daily with the amount of 3% shrimp body weight. The amount of sucrose and sugar bagasse added with feed was 190% and 196% of the feed additive, respectively, which were calculated by the formula of Avnimelech (1999) to reach a C/N ratio of 15. Each strain was added to the water body to a final concentration of 10^6^ CFU/ml at the beginning of the experiments. All experiments were carried out with zero water changes, and freshwater was added as needed to compensate for evaporation and sampling losses. All the culture trials were repeated in a subsequent replication.

**Table 1:** Experimental group setup.

Group	M13 + S	M13 + B	M15 + S	M15 + B	M17 + S	M17 + B
Sucrose	+	−	+	−	+	−
Sugar bagasse	−	+	−	+	−	+
Strains	M13	M13	M15	M15	M17	M17

### The biofloc parameters

The biofloc volume (BFV) was monitored every 3 days by using 1 l Imhoff cones after a settlement period of 30 min. The settled volume of biofloc was then noted down from the Imhoff cones reading (Avnimelech, 2007; Avnimelech et al., 2009). At the end of the experiment, samples of water were harvested for detecting the particle distribution using a Bettersize BT-93OOH laser particle size distribution analyzer (Bettersize Instruments Ltd., Liaoning, China; Gao et al., 2018).

### The shrimp growth parameters

After the experiment, vernier calipers and electronic balances were used to measure the biological body length and body weight of the shrimps, while the number of the shrimp was recorded, and assessed for the growth parameters including weight gain (WG), feed conversion ratio (FCR), feed efficiency ratio (FER), and specific growth rate (SGR) as follows.


$$\begin{array}{l} \mathrm{WG}\left(\%\right)=\left(W_{f}-W_{i}\right)\times 100/W_{i},\mathrm{FCR}=\\ \mathrm{Feed given}\;\left(W_{d}\right)/\mathrm{body weight gain}\;\left(W_{w}\right),\\ \mathrm{FER}=1/\mathrm{FCR},\mathrm{SGR}\;\left(\%\right)=\left[\begin{array}{l} \ln\;\left(W_{f}\right)-\\ \ln\;\left(W_{i}\right)/N \end{array}\right]\times 100. \end{array}$$

Where W_f_ = final weight, W_i_ = initial weight, W_d_ = dry weight, W_w_ = wet weight, ln = natural log and N = number of culture days (Irshad et al., 2016).

### Determination of water quality parameters

Water samples were collected every 3 days from each tank and filtered through 0.45 μm G F/C filter paper under vacuum pressure. Half of the water samples were analyzed spectrophotometrically for ammonium-nitrogen (NH_4_-N) and nitrite nitrogen (NO_2_^−^–N) using a CleverChem automatic discontinuous chemical analyzer (DeChem-Tech.GmbH).

### Data analysis

SPSS Statistics software (SPSS Inc. version 26.0) was used to conduct the statistical analysis. One-way ANOVA and Duncan’s multiple comparisons of the means were used to determine the differences among the treatment groups. All graphics were generated using the GraphPad Prism 6.01 software, and the findings were expressed as mean ± standard error (SD) and the differences were considered significant at the *p* < 0.05.

## Results

### Screening of bioflocculant-producing bacteria

Eighty-six cellulolytic bacteria strains that were isolated from the marine environment in the previous study were selected for flocculating activity assay (Ren et al., 2021). Thirty strains were determined to have flocculating activity over 30% (Table 2). The 10 strains with flocculating activity exceeding 80% were continuously tested for three generations to verify the genetic stability of bioflocculant-producing. Finally, three bacteria strains (M15, M13, and M17) were selected for further study for their stable bioflocculant-producing ability (Table 3).

**Table 2:** The flocculation activity of 30 strains of marine bacteria.

Strain ID	Flocculation rate (%)	Number of strains	Flocculation rate (%)
M15	93.46 ± 2.87	C51	72.37 ± 2.39
C50	86.30 ± 2.44	C64	71.98 ± 7.25
M13	85.40 ± 4.63	M20	69.91 ± 3.98
C5	84.55 ± 3.04	C88	69.76 ± 5.44
C70	82.47 ± 4.42	C67	65.91 ± 9.90
M17	82.44 ± 10.58	C26	65.37 ± 4.82
C40	82.36 ± 0.70	Z1	62.53 ± 8.72
C34	82.13 ± 5.33	C21	59.85 ± 4.34
C28	81.37 ± 1.25	C61	59.16 ± 2.72
C29	80.18 ± 1.40	C18	56.06 ± 4.54
M5	79.44 ± 8.34	C78	52.23 ± 0.46
C49	79.42 ± 2.91	C62	49.62 ± 14.34
C8	77.87 ± 2.65	Z2	43.81 ± 5.49
C66	77.23 ± 1.28	Z4	36.61 ± 9.83
C6	77.23 ± 1.28	M4	30.71 ± 9.78

Each value represents a mean ± SD (*n* = 3).

**Table 3:** Genetic stability of 10 flocculant-producing marine bacteria.

Number of strains	Generation 1	Generation 2	Generation 3
M15	95.16 ± 1.88	92.63 ± 2.86	93.46 ± 2.87
M13	89.17 ± 4.63	85.40 ± 2.63	86.40 ± 4.33
M17	92.44 ± 2.58	82.14 ± 3.58	84.44 ± 1.58
C5	84.55 ± 3.04	78.51 ± 2.02	_
C28	81.37 ± 1.25	76.10 ± 1.25	–
C29	80.18 ± 1.40	–	–
C34	82.13 ± 5.33	78.13 ± 3.33	–
C40	82.36 ± 0.70	64.73 ± 0.70	49.50 ± 0.90
C50	86.30 ± 2.44	69.84 ± 2.34	66.84 ± 2.34
C70	86.73 ± 4.42	82.47 ± 4.22	56.30 ± 3.42

Each value represents a mean ± SD (*n* = 3) and –: No flocculation activity.

### Identification and characterization of bioflocculant-producing bacteria

Molecular analysis of the 16S rDNA sequences results showed that the strains M15, M13, and M17 were *Bacillus altitudinis*, *Bacillus pumilus*, and *Bacillus cereus*, respectively (Table 4). The nucleotide sequence of the 16S rDNA sequences had been submitted to GenBank and assigned accession number ON870799(M13), ON870800(M15), and ON870801(M17). The Biolog GEN III assay results also confirmed that all three strains belonged to the *Bacillus* sp. The strains M13 and M17 had identical results compared to 16S rDNA analysis. The M15 strain which was identified as *B. altitudinis* was recognized as *B. pumlius/safensis* because there is no *B. altitudinis* information in the Biolog database and the system gives the closest strain instead (Table 5). The details of Biolog GEN III plate data are presented in the Supplementary material (Supplementary Figure S1; Supplementary Table S1).

**Table 4:** The results of 16S rDNA identification.

Number of strains	Identification result	Similarity
M13	*Bacillus altitudinis*	100%
M15	*Bacillus pumilus*	99.87%
M17	*Bacillus cereus*	99.93%

**Table 5:** The identification results of 3 strains by Biolog system.

Number of strains	Standard strain	PROB	DIST	SIM
M13	*Bacillus pumilus/safensis*	0.953	6.568	0.555
M15	*Bacillus pumilus/safensis*	0.956	4.006	0.687
M17	*Bacillus cereus/thuringiensis*	0.861	5.329	0.631

### Cellulolytic activity and ability of sugarcane bagasse utilization

**Figure 1:**
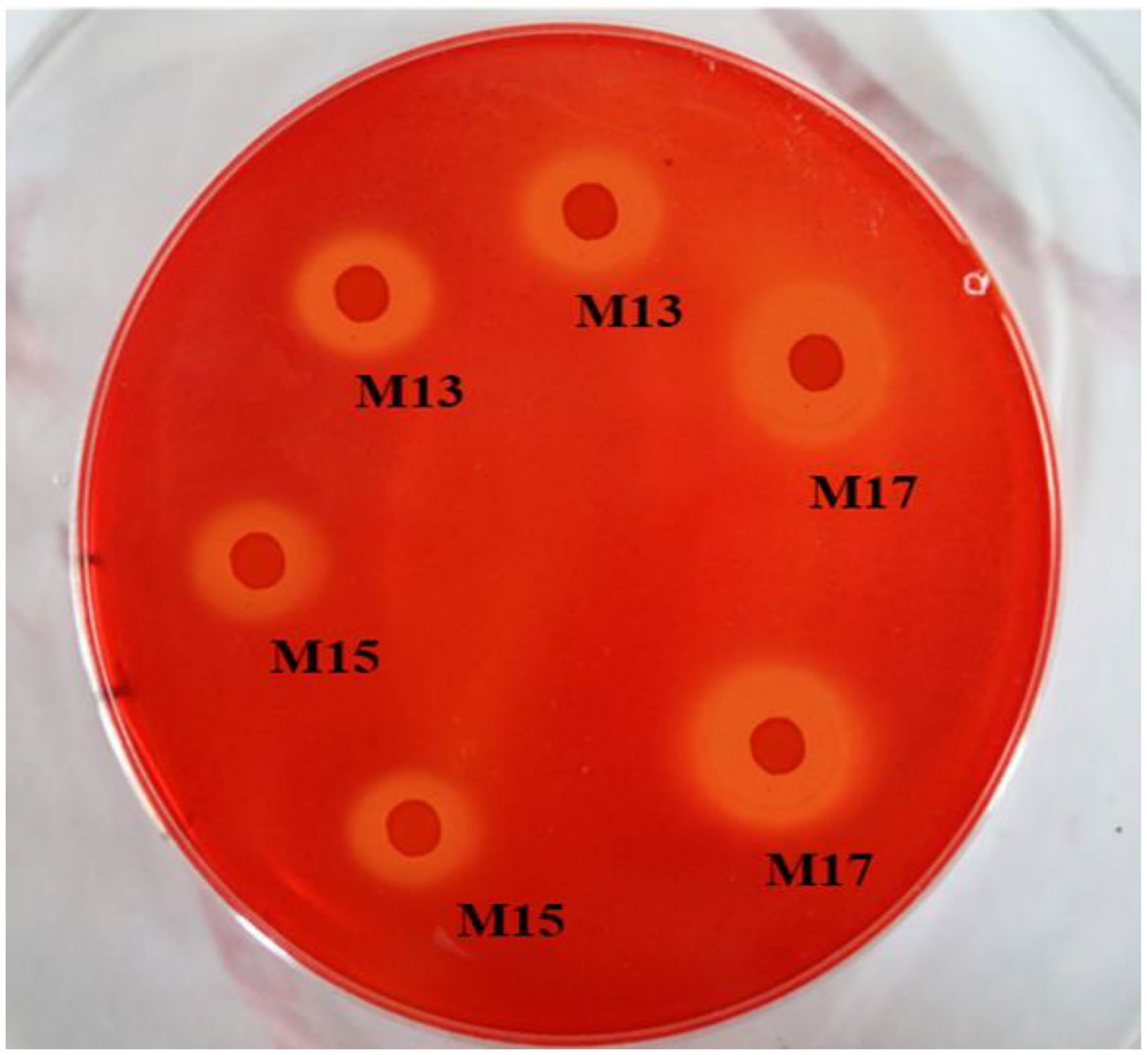
Cellulase hydrolysis circle of the three strains. The surrounding clear zone of the colonies indicated the ability of the bacteria to consume CMC on the plates.

**Figure 2:**
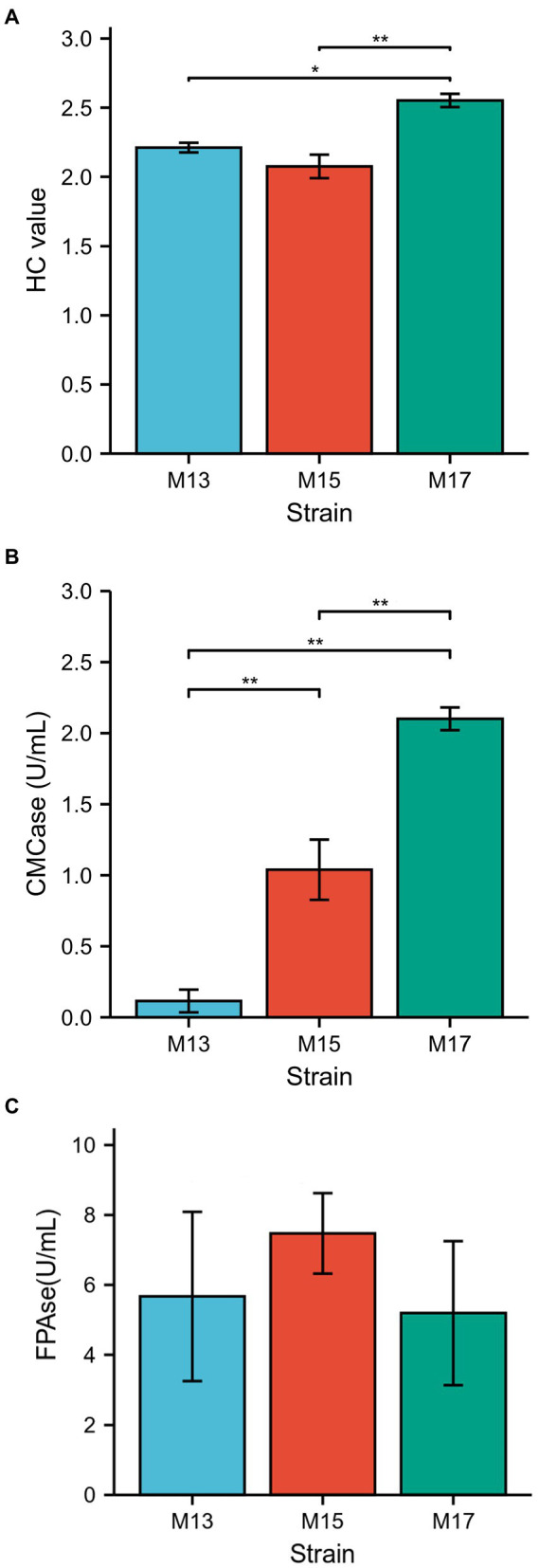
Comparison of the cellulolytic activity of the three strains. **(A)** H/C ratio, **(B)** CMCase, and **(C)** FPAse.

All three strains showed clear zones around the bacterial colonies after being stained with Congo red on CMC agar plates (Figure 1). Strain M17 had the largest hydrolysis capacity (HC) value at 2.55 ± 0.06, and HC values of M13 and M17 were 2.21 ± 0.03 and 2.08 ± 0.1, respectively (Figure 2A). Also, strain M17 showed the highest CMCase activity (2.101 ± 0.08) in the culture supernatant, and the CMCase activity of M13 was significantly lower (Figure 2B). All the strains possessed FPase activity with no significant difference (Figure 2C).

**Figure 3:**
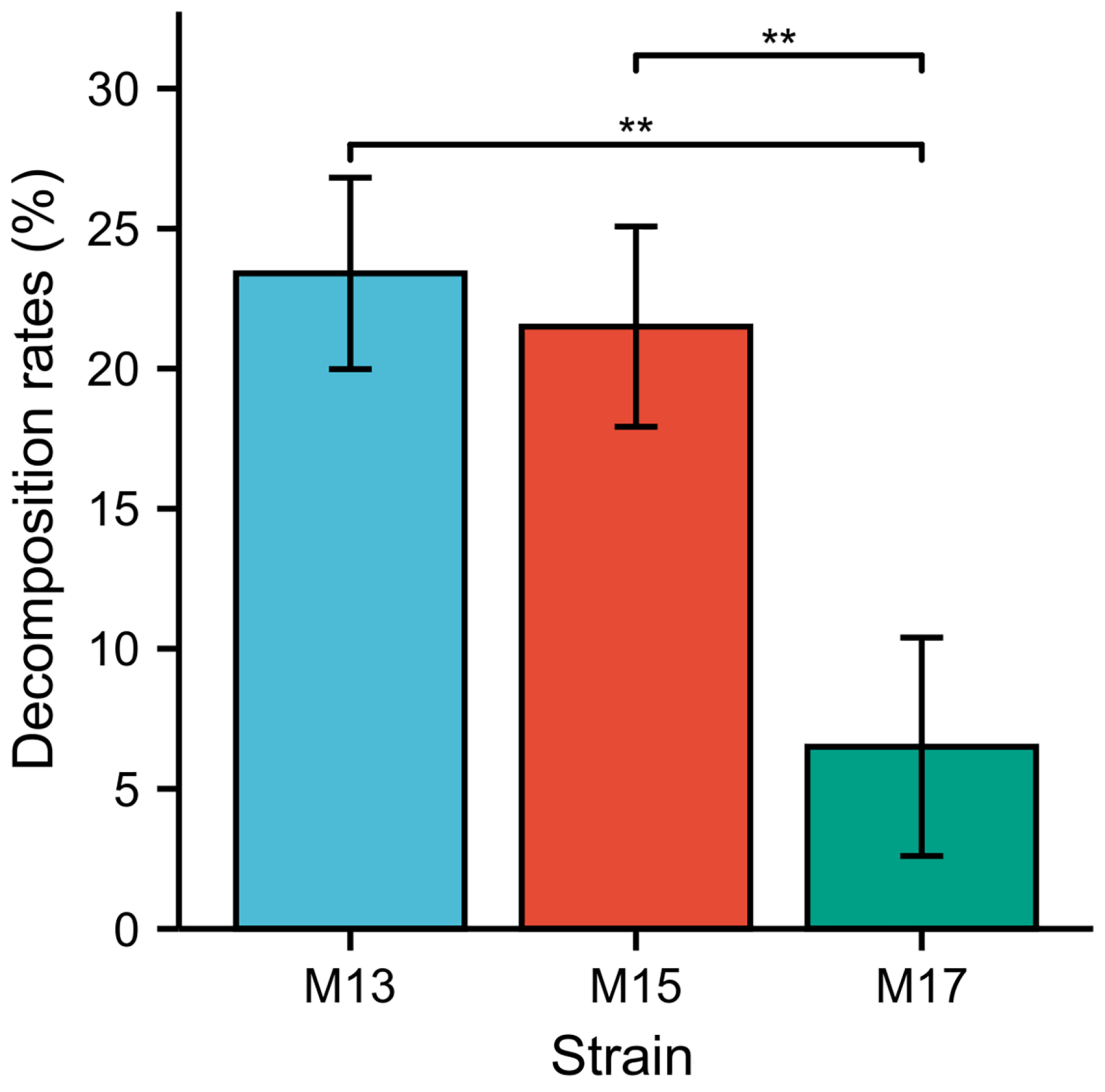
The decomposition rate of SB by the three strains. **p < 0.01.

The sugarcane bagasse decomposition experiment results showed that strains M13 and M15 could decompose 24% and 22% SB in 3 days, respectively, while strain M17 had the lowest decomposition rate which is 7% in 3 days (Figure 3).

### The biofloc parameters

**Figure 4:**
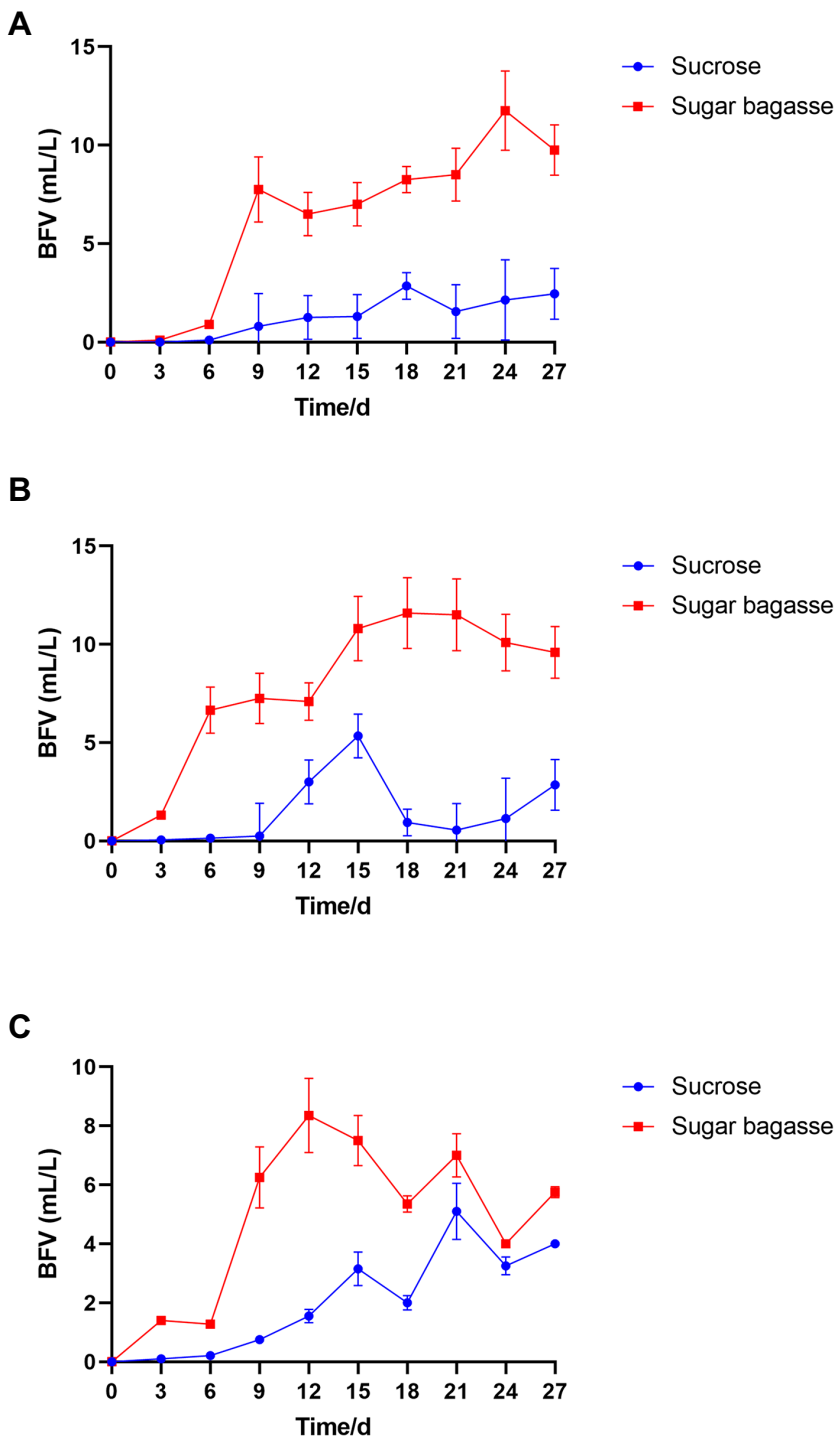
Effects of different carbon sources on BFV with each strain. **(A)** Strain M13, **(B)** Strain M15, and **(C)** Strain M17.

After the M13, M15, and M17 strains were added to shrimp culture tanks with different carbon sources (Table 1), the biofloc system was monitored for 27 days. As shown in Figure 4, all the groups with the sugarcane bagasse have formed biofloc quicker and higher than the sucrose groups. The biofloc volume in the system contained SB and strain M13 raised on day 6 and reached a high level on day 9, while the BFV in sucrose groups started to rise on day 9 and maintained at a low level until the end of the experiment (Figure 4A). In the strain M15 and M17 groups, SB promoted BFV 3 days after the experiment started, while in sucrose groups, biofloc started to accumulate after 9 days, and only strain M17 could promote BFV with sucrose close to SB group after 21 days (Table 6; Figures 4B,C).

**Table 6:** The BFV data of 3 strains.

Time/day	M13		M15		M17	
	Sucrose	Sugar bagasse	Sucrose	Sugar bagasse	Sucrose	Sugar bagasse
0	0	0	0	0	0	0
3	0.01 + 0.02	0.11 + 0.02	0.05 + 0.01	1.33 + 0.12	0.11 + 0.02	1.40 + 0.15
6	0.11 + 0.20	0.91 + 0.19	0.15 + 0.01	6.65 + 1.19	0.21 + 0.02	1.28 + 0.13
9	0.80 + 1.67	7.75 + 1.65	2.95 + 0.62	7.25 + 1.28	0.75 + 0.06	6.25 + 1.04
12	1.25 + 1.11	6.50 + 1.10	5.25 + 1.16	7.10 + 0.95	1.55 + 0.23	8.35 + 1.26
15	1.30 + 1.11	7.00 + 1.10	0.90 + 0.05	10.80 + 1.64	3.15 + 0.57	7.50 + 0.86
18	2.85 + 0.68	8.25 + 0.67	0.50 + 0.07	11.60 + 1.81	2.00 + 0.24	5.35 + 0.28
21	1.56 + 1.36	8.50 + 1.35	0.55 + 0.09	11.50 + 1.84	5.10 + 0.95	7.00 + 0.73
24	2.15 + 2.04	11.75 + 2.02	1.15 + 0.23	10.10 + 1.44	3.25 + 0.31	4.00 + 0.00
27	2.45 + 1.30	9.75 + 1.28	3.15 + 0.57	9.60 + 1.32	4.00 + 0.12	5.75 + 0.18

**Figure 5:**
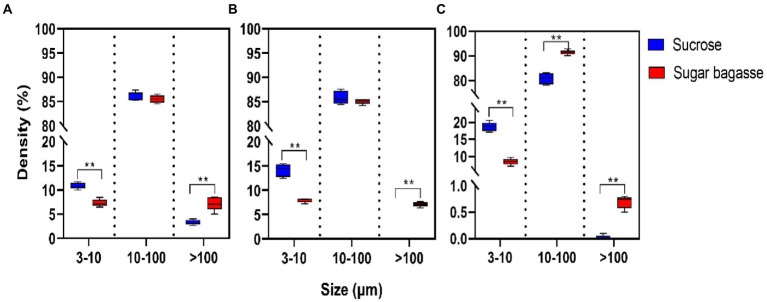
Effects of different carbon sources on the particle size distribution of bio floc. **(A)** Strain M13, **(B)** Strain M15, and **(C)** Strain M17.

The particle size distribution of the biofloc was influenced by both the strain and the carbon sources input into the system. The largest proportion of the particles consisted of medium-sized particles (10–100 μm). In the M13 and M15 groups, the percentage of medium-sized particles was around 85% with no significant between the two carbon sources (Figures 5A,B). In the M17 groups, 91.45% of particles were medium-sized when SB was added into the system and the same size percentage was 80.85% when sucrose was used (Figure 5C). The sucrose had promoted the small-sized particles (3–10 μm) formation while the SB addition had stimulated the development of large-sized particles (>100 μm) in all the strain groups (Figure 5).

### The shrimp growth parameters

**Figure 6:**
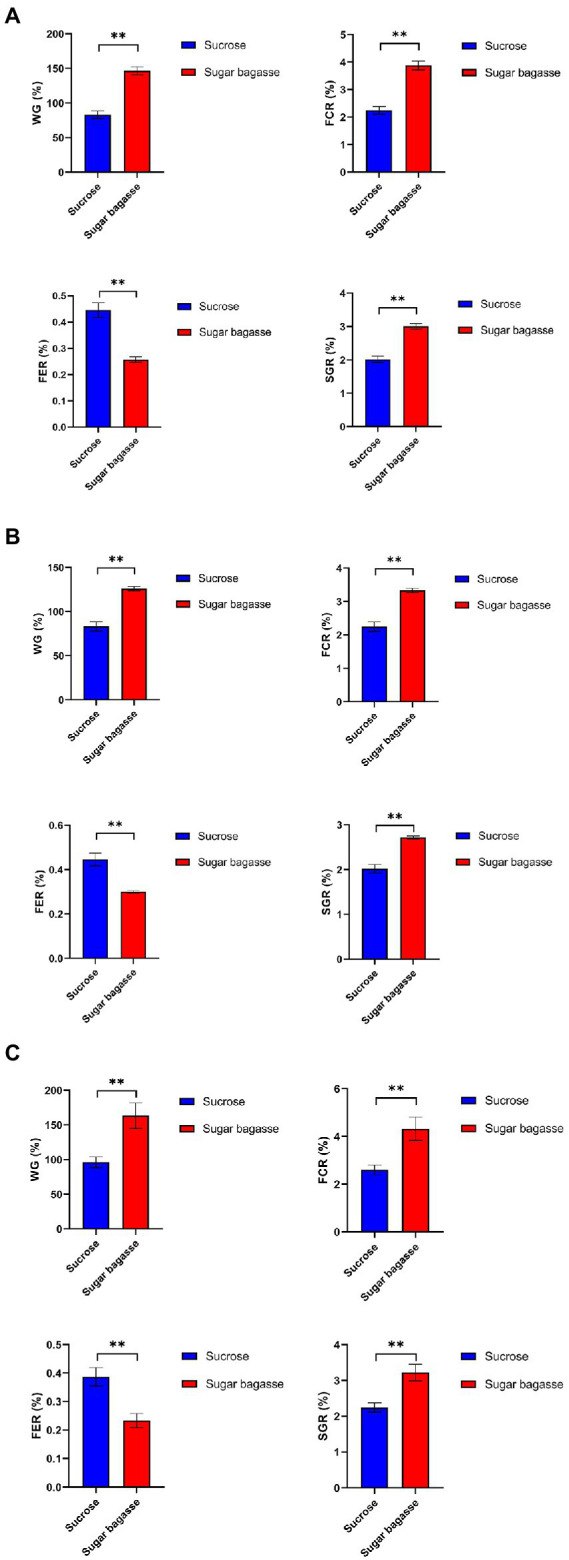
Growth performance of shrimps with different strains. **(A)** M13, **(B)** M15, and **(C)** M17.

The growth performance of shrimp in different treatments is shown in Figure 6. In the M13 strain group, the SB addition, compared to sucrose addition, significantly increased the WG, FCR, and SGR of the shrimps by 66.2%, 0.99%, and 1.63%, respectively, and reduced the FER by 0.19% (Figure 6A). The results of M15 and M17 strains also showed that SB had a positive effect on shrimp growth (Figures 6B,C).

### Water quality parameters

**Figure 7:**
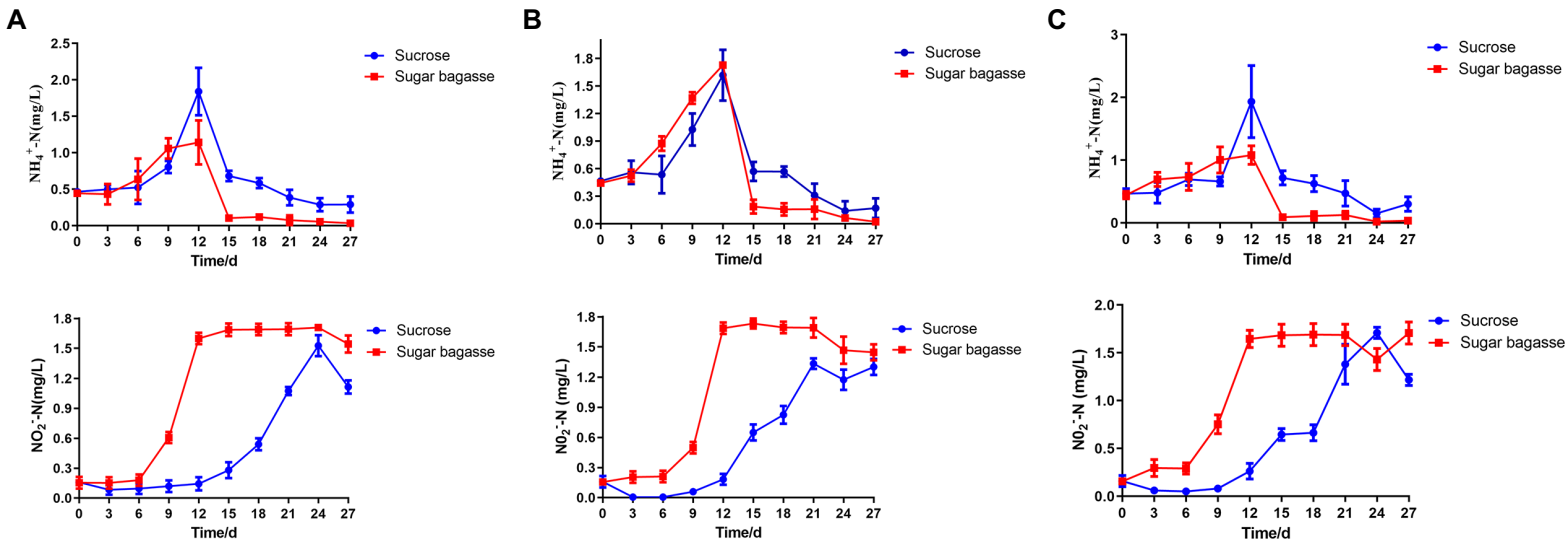
Changes in water quality parameters of BFT system with different strains. **(A)** M13, **(B)** M15, and **(C)** M17.

During the 27 days culture period, the concentration of ammonium-nitrogen (NH_4_-N) increased gradually and peaked on the 12th day. With the addition of SB, all the three strains could decrease NH_4_-N more quickly than sucrose addition and maintained at a relatively low level (Figure 7). The concentration of nitrite nitrogen (NO_2_-N) raised over time and stayed at a high level after 12 days when using SB as a carbon source. However, NO_2_-N of each sucrose group increased slowly and reached the highest level after 21 or 24 days (Figure 7).

## Discussion

Because of their biodegradability, innocuity, safety to humans, and environmental friendliness, biofloculants have attracted more attention in aquaculture and wastewater treatment systems than chemical flocculants (Okaiyeto et al., 2020). Microorganisms that can produce different kinds of bioflocculant distribute widely in both soil and water environments. Many efforts have been made to isolate bioflocculant-producing bacteria which were identified in different species, such as *B. subtilis* (Jiang et al., 2019), *B. megaterium* (Luo et al., 2016), *B. licheniformis* (Chen et al., 2017b), *Klebsiella* sp. (Ma et al., 2019), *Pseudomonas* sp. (Qi et al., 2019), *Alteromonas* sp. (Chen et al., 2017a), and *Halobacillus* sp. (Cosa et al., 2013). *Bacillus* has been used extensively as a probiotic in aquaculture to improve feed utilization, stress response, immune response, and disease resistance of aquatic animals (Kuebutornye et al., 2019). The sporulation capacity and non-pathogenic character make *Bacillus* sp. bioflocculant-producing excellent candidates to apply in the biofloc aquaculture system. In this work, three bacteria strains with high flocculating activity determined by kaolin clay suspension assay were isolated from previously constructed Microorganisms Collection of South China Sea, MCSCS (Ren et al., 2021). The flocculating efficiency activity of strain M15 was the highest and stablest (92.63%–95.16%) according to the experiment results (Table 3), which is similar to the high bioflocculating strains from previous studies, e.g., *Klebsiella* sp. OS-1(95%) and *Pseudomonas* sp. HP2 (92.5%; Ma et al., 2019; Qi et al., 2019). The strain M13 and strain M17 exhibited lower flocculating efficiency but they were still over 80% (82.14%–92.44%). All the three strains could maintain the high ability to aggregate kaolin clay suspension after continuous cultured for generations compared to other strains, which made them suitable for long-term biofloc culture system.

The 16S rDNA sequence alignment analysis and Biolog GEN III Microplate assay results all suggested they belong to the *Bacillus* sp. (Tables 4, 5). There have been several studies on *B. cereus* as bioflocculant-producing strains in water treatment and microalgae harvesting (Sarang and Nerurkar, 2020; Zhang S. et al., 2021; Zhang Y. et al., 2021). In this work, strain M17 was also found closely related to *B. cereus*, which might have the same potential applications as previous reports. The carbon source metabolic characteristics of *B. altitudinis* does not exist in the Biolog database, so the GEN III Microplate assay identified strain M13 as *B. pumilus/safensis*, the same as strain M15 but they still differed in carbon source metabolic abilities (Supplementary Figure S1; Supplementary Table S1). The *B. altitudinis* and *B. pumilus* strains were found useful in antimicrobial activity, stress tolerance, and plant growth-promoting (Li et al., 2020; Shah et al., 2021), but there were few reports on their bioflocculant-producing ability and application in wastewater treatment.

The cellulolytic activity assay also confirmed all three strains could digest carboxymethylcellulose (CMC) and filter paper (cellulose; Figures 1, 2) since they were identified as cellulolytic bacteria in the previous study (Ren et al., 2021). However, the sugarcane bagasse decomposition results showed that strain M13, which exhibited the lowest CMCase activity of the three strains, had the highest SB decomposition rate and strain M17 had the opposite results (Figure 3). These results might due to the complex composition of SB which could not be hydrolyzed thoroughly only by cellulase (Alokika et al., 2021). Our study also suggests that strain M13 has more enzymes to utilize different kinds of carbon sources, especially with complex compositions.

To maintain an appropriate C/N ratio for the biofloc to form and continue to work, many carbon sources have been applied to the aquaculture system. Small molecule compounds, e.g., glucose, acetate, and glycerol, could be used by bacteria quickly in the biofloc system (Crab et al., 2009), but their prices are too high to apply on large scale. Sucrose is also an effective carbon source for biofloc-based shrimp culture systems (Guo et al., 2022). In this study, we investigated the feasibility of applying sugarcane bagasse, the byproduct of the sucrose industry, as a substitute for sucrose in biofloc technology (BFT) systems. Polysaccharides, such as all kinds of the starch-rich compound and their enzyme-hydrolyzed products have already been studied in the BFT system to reduce the cost of extra addition other than feeds (Shang et al., 2018; Panigrahi et al., 2019). Solid-phase biodegradable polymers (BDPs), such as Polyhydroxybutyrate (PHB) and polycaprolactone (PCL), could be used as carbon sources as well as biofilm carriers for bacteria to aggregate (Liu et al., 2019). SB, a low-cost insoluble polysaccharide, is a promising alternative carbon source for BFT. To overcome the disadvantage of hard to degrade, we also screened SB degradable bioflocculant-producing bacteria to add to the system simultaneously. The results showed that, compared to sucrose, SB with the three selected strains could promote the biofloc and the growth of shrimp at the same time. The BFV of the groups supplied with SB started to raise after 3 or 6 days and maintained at a high level from the day 12 to the end of our experiment, whereas the BFV of sucrose groups kept at a low level with strains M13 and M15 and only strain M17 promoted BFV close to SB group at the end (Figure 4). Rich in protein, lipid, and other nutrition, the bioflocs can be considered a complementary food source for cultured shrimps or fish, which could reduce the feed cost by lower the FER (Gallardo-Collí et al., 2019). The large-size biofloc (>100 μm) contained the highest levels of protein and lipid (Ekasari et al., 2014). In our study, the SB addition increased the ratio of the large-size biofloc significantly (Figure 5), which lead to a high weight gain (WG) and specific growth rate (SGR) of the shrimp (Figure 6). One of the important functions of BFV is water quality purification. Since ammonia (NH_3_-N) and nitrite (NO_2_) are highly toxic for the animals in water (Putra et al., 2020), we paid special attention to these two nitrogenous wastes which are mainly derived from the high protein feed. as shown in Figure 7, the concentration of NH_3_-N of different groups all raised until day 12 and started to decline, but the high points of strain M13 and M17 with SB were lower than the sucrose groups. All SB groups had better ammonia removal rates compared to sucrose groups. The nitrite accumulated in all tanks as the continuous feed input and no water exchange but slowly in the tanks containing sucrose. These results suggested that M13, M15, and M17 do not possess a strong denitrification ability and sucrose might facilitate other denitrifying bacteria growth in the water. To achieve a better result of nitrite nitrogen removal, other aerobic denitrification bacteria strains or microalgae could be added to the biofloc system for further study.

## Conclusion

In conclusion, we screened three bioflocculant and cellulase-producing Strains, M13(*B. altitudinis*), M15(*B. pumilus*), and M17(*B. cereus*), with the ability to promote biofloc formation by using sugarcane bagasse as a carbon source. When applied these strains into the BFT system with SB as a replacement carbon source of sucrose, the biofloc, and shrimp growth have been improved, and the NH_4_-N concentration has been controlled under zero water change. A low-cost and high-efficient BFT system for *L. vannamei* has been preliminarily established in our study.

## Data availability statement

The data presented in the study are deposited in the GenBank repository, accession number ON870799, ON870800, and ON870801.

## Author contributions

JW and HL designed the study. YC, XZ, XC, and AH analyzed the data. JW, HL, YC, and ZX wrote and revised the manuscript. JW, XX, WR, and YZ performed the experiments. All authors contributed to the article and approved the submitted version.

## Funding

This study was supported financially by the Hainan Province Science and Technology Special Fund (ZDYF2020095 and ZDYF2018109), Natural Science Foundation of Hainan Province (2019RC106), National “13th Five-Year Plan” Marine Economic Innovation Development Demonstration City Project (HHCL201813 and HHCL201802), Natural Science Foundation of China (32060835), and Scientific Research Foundation of Hainan University (KYQD(ZR)1819).

## Conflict of interest

The authors declare that the research was conducted in the absence of any commercial or financial relationships that could be construed as a potential conflict of interest.

## Publisher’s note

All claims expressed in this article are solely those of the authors and do not necessarily represent those of their affiliated organizations, or those of the publisher, the editors and the reviewers. Any product that may be evaluated in this article, or claim that may be made by its manufacturer, is not guaranteed or endorsed by the publisher.
