# Supplementary figures and images for: Screening of bioflocculant and cellulase-producing bacteria strains for biofloc culture systems with fiber-rich carbon source
Source: Front Microbiol. 2022 Nov 24;13:969664. doi: 10.3389/fmicb.2022.969664 (PMC9729547; doi:10.3389/fmicb.2022.969664)

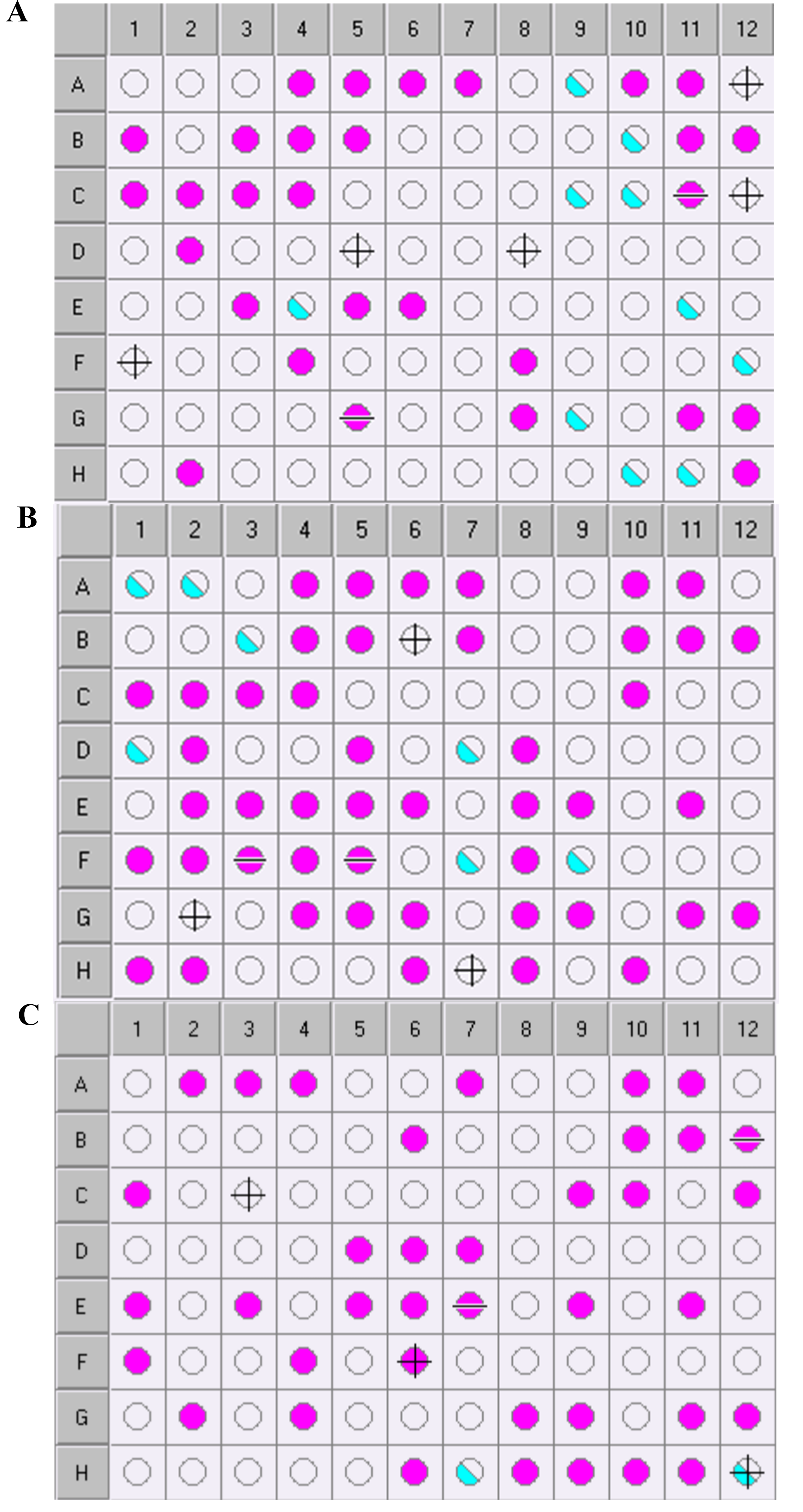

Supplement: Supplementary file 1 [file Image_1.tif]
