# Supplementary material for: Screening of bioflocculant and cellulase-producing bacteria strains for biofloc culture systems with fiber-rich carbon source
Source: Front Microbiol. 2022 Nov 24;13:969664. doi: 10.3389/fmicb.2022.969664 (PMC9729547; doi:10.3389/fmicb.2022.969664)
Supplement: Supplementary file 2 [file Table_1.docx]

Table S1 The utilization of 94 different substrates in Biolog-GEN III plate by 3 strains.

| Number | Substrate | M13 | M15 | | M17 | Number | Substrate | M13 | M15 | M17 |
| --- | --- | --- | --- | --- | --- | --- | --- | --- | --- | --- |
| A1 | Contrast | − | | − | − | E1 | Glycogen | − | / | + |
| A2 | Dextrin | − | | / | /// | E2 | Glycyl-L-proline | − | + | − |
| A3 | D-Maltose | − | | − | /// | E3 | L-Alanine | + | + | + |
| A4 | D-Trehalose | + | | + | + | E4 | L-Arginine | / | + | / |
| A5 | D-Cellobiose | + | | + | − | E5 | L-Aspartic Acid | + | + | + |
| A6 | Gentiobiose | + | | + | − | E6 | L-Glutamic Acid | + | + | + |
| A7 | Sucrose | + | | + | + | E7 | L-Histidine | − | − | // |
| A8 | D-Turanose | − | | − | − | E8 | L-Pyroglutamic Acid | − | + | − |
| A9 | Stachyose | / | | − | − | E9 | L-Serine | − | + | // |
| A10 | Positive Control | + | | + | + | E10 | Lincomycin | − | − | − |
| A11 | pH 6 | + | | + | + | E11 | Guanidine HCl | / | /// | + |
| A12 | pH 5 | /// | | − | + | E12 | Niaproof 4 | − | − | − |
| B1 | D-Raffinose | + | | − | − | F1 | Pectin | /// | + |  |
| B2 | α-D-Lactose | − | | − | − | F2 | D-Galacturonic Acid | − | + | / |
| B3 | D-Melibiose | + | | / | − | F3 | L-Galactonic Acid lactone | − | // | − |
| B4 | β-Methyl-D-glucoside | + | | + | − | F4 | D-Gluconic Acid | + | + | // |
| B5 | D-Salicin | + | | + | − | F5 | D-Glucuronic Acid | − | // | + |
| B6 | N-Acetyl-D-glucosamine | − | | + | − | F6 | Glucuronamide | − | − | / |
| B7 | N-Acetyl-β-D-mannosamine | − | | + | / | F7 | Mucic Acid | − | / | − |
| B8 | N-Acetyl-D-galactosamine | − | | − | − | F8 | Quinic Acid | + | + | − |
| B9 | N-Acetylneuraminic Acid | − | | − | − | F9 | D-Saccharic Acid | − | / | − |
| B10 | 1% NaCl | / | | + | + | F10 | Vancomycin | − | − | − |
| B11 | 4% NaCl | + | | + | + | F11 | Tetrazolium violet | − | − | − |
| B12 | 8% NaCl | + | | + | // | F12 | Tetrazolium blue | / | − | − |
| C1 | α-D-Glucose | + | | + | − | G1 | ρ-Hydroxy-phenylacetic Acid | − | − | − |
| C2 | D-Mannose | + | | + | − | G2 | Methyl pyruvate | − | /// | + |
| C3 | D-Fucose | + | | + | /// | G3 | D-Lactic Acid methyl ester | − | − | − |
| C4 | D-Galactose | + | | + | − | G4 | L-Lactic Acid | − | + | + |
| C5 | 3-Methyl-glucose | − | | − | − | G5 | Citric Acid | // | + | − |
| C6 | D-Fructose | − | | − | − | G6 | α-Keto glutaric Acid | − | + | + |
| C7 | L-fucose | − | | − | − | G7 | D-Malic Acid | − | − | − |
| C8 | L-Rhamnose | − | | − | − | G8 | L-Malic Acid | + | + | + |
| C9 | Inosine | / | | + | + | G9 | Bromo-Succinic Acid | / | + | / |
| C10 | 1% sodium lactate | / | | + | + | G10 | Nalidixic Acid | − | − | − |
| C11 | Fusidic Acid | // | | − | − | G11 | Lithium chloride | + | + | + |
| C12 | D-serine | /// | | − | + | G12 | Potassium tellurite | + | + | + |
| D1 | D-Sorbitol | − | | − | − | H1 | Tween 40 | − | + | / |
| D2 | D-Arabitol | + | | + | − | H2 | γ-Amino butyric Acid | + | + | − |
| D3 | D-Mannitol | − | | − | − | H3 | α-Hydroxy-butyric Acid | − | − | + |
| D4 | Myoinositol | − | | − | − | H4 | β-Hydroxy-D,L butyric Acid | − | − | / |
| D5 | Glycerol | /// | | + | / | H5 | α-Keto butyric Acid | − | − | / |
| D6 | D-Glucose-6-phosphate | − | | − | + | H6 | Acetoacetic Acid | − | + | + |
| D7 | D-Fructose-6-phosphate | − | | / | + | H7 | Propionic Acid | − | − | + |
| D8 | D-Aspartic Acid | /// | | + | − | H8 | Acetic Acid | − | + | + |
| D9 | D-Serine | − | | − | / | H9 | Formic Acid | − | − | + |
| D10 | Troleandomycin | − | | − | − | H10 | Aztreonam | / | + | + |
| D11 | Rifamycin SV | − | | − | − | H11 | Sodium butyrate | / | + | + |
| D12 | Minocycline | − | | − | − | H12 | Sodium bromate | + | − | / |

Note. +: positive response; −: negative response; /: borderline; //: mismatched positive; and ///: mismatched negative.
